# The Occurrence of a Negative Energy Balance in Holstein-Friesian and Simmental Cows and Its Association with the Time of Resumption of Reproductive Activity

**DOI:** 10.3390/metabo12050448

**Published:** 2022-05-17

**Authors:** Krzysztof Młynek, Ilona Strączek, Beata Głowińska

**Affiliations:** 1 Faculty of Agrobioengineering and Animal Sciences, Institute of Animal Science and Fisheries, Siedlce University of Natural Sciences and Humanities, 08-110 Siedlce, Poland; ilona.straczek@uph.edu.pl; 2 Faculty of Animal Breeding and Biology, Department of Animal Physiology and Physiotherapy, Bydgoszcz University of Science and Technology, 85-084 Bydgoszcz, Poland; beata.glowinska@pbs.edu.pl

**Keywords:** dairy cows, energy balance, reproduction activity, biomarkers, breed

## Abstract

Intensive lactation (lactogenesis) in cows is conducive to a negative energy balance (NEB), so the search for traits associated with the physiological capacity to cope with its consequences is a current area of research. This is especially important because NEB overlaps with the resumption of the reproductive cycle, which determines the profitability of herds. This study analysed the relationship between NEB and the time of resumption of reproductive activity in cows with varying genetic potential (Simmental and Holstein-Friesian), fed a similar diet (TMR). The aim of the study was to analyse the dependencies between NEB markers and changes in progesterone levels between 25 and 31 days postpartum. A strong positive correlation was shown between daily milk production (DMP) and loss of body condition (LBCS; 0.772; *p* ≤ 0.05). These parameters were associated with the levels of NEB biomarkers. Higher values of NEB indicators (LBCS, C16:0, C18:1, NEFA, and BHBA) were usually noted during periods with higher DMP (II and III). The trends observed were confirmed by positive correlation coefficients (r), which ranged from 0.324 to 0.810 (*p* ≤ 0.05). The reverse trend was noted for glucose and leptin, which decreased as productivity increased, as confirmed by r values from −0.368 to −0.530 (*p* ≤ 0.05). In both breeds, the glucose and leptin levels decreased as DMP increased. Higher values for NEB indicators were shown to be negatively correlated with progesterone levels (r from −0.300 to −0.712; *p* ≤ 0.05), and a lower progesterone level was associated with a longer calving-to-first-service interval and calving-to-conception interval. The rate of postpartum triglyceride release depends on daily milk production, and therefore the adaptability of the liver should be considered an important element of mitigation of the consequences of NEB. This may have practical applications by extending productive life, which is often shortened due to deteriorating reproductive performance.

## 1. Introduction

The transition period is an important and somewhat unstable period of the production cycle of dairy cows. At this time, systemic mobilization of tissues takes place, and one of the consequences of the metabolic and humoral changes is a decrease in appetite [1,2]. This affects systemic homeostasis in cows, including reproductive performance and even milk production in the subsequent lactation [3,4]. In the case of reproductive traits, it is significant that regeneration following parturition overlaps with increasing lactation and the energy deficit, which can impair reproduction and affect activation of the ovaries after parturition [5,6,7,8,9]. During the transition period, extensive metabolic and endocrine changes lead to a decrease in dry matter intake, a disturbance of energy homeostasis, and a negative energy balance [10]. Metabolic dysfunctions of the transition period are mainly due to rapid lipolysis [9]. The loss in body condition (BCS) observed at this time is due in part to the role of leptin, one of the biomediators on pathways of energy metabolism, including lipolysis [11,12]. This mechanism increases the share of non-esterified fatty acids (NEFAs) in the body, and the increasing lipolysis causes retention of triglycerides in the hepatocytes [13]. The liver, as an organ coordinating extensive metabolic changes, is involved in oxidation of fatty acids and glycogenesis and reduces the level of triacylglycerols [14,15]. Excessive triacylglycerol levels impair its metabolism and increase BHBA production [1]. An increased NEFA level in the blood can adversely affect oocyte development and reproductive performance [16]. In addition, a persistent elevated BHBA level negatively affects the glucose level in the body [17]. 

Fertility is an important factor determining the profitability of dairy cattle herds. Fertility disorders are associated with expenditures for treatment, repeated insemination, and even culling [18,19,20]. Metabolic dysfunctions induced by NEB are therefore a serious challenge for dairy producers, especially as the global trend of increasing productivity of dairy herds still persists. Therefore, studies on the consequences of NEB, including for fertility, remain highly relevant. The aim of this study was to assess the relationship between NEB and the time of the first reproductive activity after calving in Holstein-Friesian and Simmental cows.

## 2. Results 

Both cow breeds showed an increase in lactation intensity between the first and second stages of lactation (SL; Table 1), which is confirmed by the difference in daily milk production (DMP), which averaged 8.9 kg/d (*p* ≤ 0.05) in the Holstein-Friesian group (HF) and 9.0 kg/d (*p* ≤ 0.05) in the Simmental group (SIM). After SL II there was a significant increase in milk production, as indicated by the differences in DMP between SL II and III: 5.3 kg/d on average in SIM and 4.5 kg/d in HF (*p* ≤ 0.05). The average body condition score (BCS) assessed 5 days before calving was 2.79 in HF and 2.83 in (SIM). In the case of HF cows, the loss in body condition (LBCS) up to SL II (0.08) was greater than the loss between SL II and III (0.02; *p* ≤ 0.05). In the group of SIM cows, the decline in body condition score (LBCS) remained similar up to SL II, but between SL II and III there was a further loss of BCS amounting to 0.03 (*p* ≤ 0.05). There was a strong positive correlation (0.772, *p* ≤ 0.05) between daily milk production and LBCS.

The proportion of C16:0 in the milk increased over the course of lactation. Between SL I and II it increased by 3.1% in the milk of HF cows and by 3.11% in the milk of SIM cows (*p* ≤ 0.05). Between SL II and III it increased by a further 2.09% in HF and by 1.94% in SIM. A similar trend, in both groups, was noted for C18:1. Up to SL II the average increase in its proportion was 1.31% in HF (*p* ≤ 0.05) and 0.9% in SIM (*p* ≤ 0.05). After this time, it increased by 0.46% in the HF group and by 0.42% in SIM, but in this case the significance of the differences was not confirmed statistically. In the HF group, no clear trends in C18:0 or C18:2 were observed. In the milk of SIM cows, however, the percentage share of C18:0 increased at a similar rate throughout the early lactation period, on average by 0.31% (*p* ≤ 0.05). The percentage share of C18:2 increased most sharply up to the second stage of lactation (SL II) (0.33%, *p* ≤ 0.05). Daily milk production (DMP) and LBCS were most strongly correlated with the proportions of C16:0 and C18:1, with correlation coefficients from 0.431 to 0.676 (*p* ≤ 0.05). Correlations with C18:0 and C18:2 were less strong, from 0.364 to 0.394 (*p* ≤ 0.05).

The level of non-esterified fatty acids (NEFA) in the blood (Table 1) was usually higher in lactation stages with higher DMP in both cow breeds. However, in HF the share of this component was usually higher. The increase in NEFA was considerable between SL I and II, as confirmed by differences of 52.2 and 58.0 mmol L^−1^ in HF and SIM, respectively (*p* ≤ 0.05). In the next interval, between SL II and III, the increase in NEFA was smaller in both breeds. The differences were 35.9 mmol L^−1^ for HF and 38.4 mmol L^−1^ for SIM (*p* ≤ 0.05). The differences in DPM between SL II and III indicate that these changes were associated with decreased lactation.

The greatest increase in the plasma level of β-hydroxybutyrate (BHBA) was shown between SL II and III (Table 1). The difference was 0.43 mmol L^−1^ in HF and mmol L^−1^ in SIM 0.89 (*p* ≤ 0.05). In the previous period (SL I–II), the corresponding increases were 0.360 and 0.389 mmol L^−1^ (*p* ≤ 0.05). Non-esterified fatty acids (NEFA) and BHBA levels were relatively strongly correlated with DPM and LBCS, as indicated by positive correlation coefficients ranging from 0.634 to 0.810 (*p* ≤ 0.05). 

During the period in which DPM was highest (SL III), levels of glucose (GLU) and leptin were lower (Table 1). In the case of GLU, the greatest reduction was noted up to SL II; the differences were 0.51 mmol L^−1^ in HF and 0.45 mmol L^−1^ in SIM (*p* ≤ 0.05). The leptin level was highest in SL I in HF and in SL II in the case of SIM. The greatest decline in the leptin level in the blood of HF cows was noted between SL I and II (0.29 ng ml^−1^; *p* ≤ 0.05). In SIM cows, however, the greatest drop in the leptin level was not noted until the period approaching the peak of lactation, between SL II and III (0.32 ng ml^−1^; *p* ≤ 0.05).

The increase in the progesterone (P4) level between 24 (P_24_) and 27 (P_27_) days postpartum (Table 2) was sharper in SIM. The difference between P4_24_ and P4_27_ was 2.12 ng/mL (*p* ≤ 0.05), while the corresponding difference in HF was 1.90 ng/mL (*p* ≤ 0.05). In the next interval (29 days postpartum—P4_29_) its level showed a marked increase, by 7.83 ng/mL in HF and 8.15 ng/mL in SIM (*p* ≤ 0.05). Between (P_29_) and 31 days postpartum (P4_31_), P4 production declined. Although the level of this hormone still showed an increasing tendency, the differences were smaller: 0.9 ng/mL in HF and 1.2 ng/mL in SIM (*p* ≤ 0.05). 

Table 3 presents the results of the analysis of negative energy balance (NEB) markers and fertility indicators depending on changes in P4 levels between 29 and 31 days postpartum (P4_29-31_). During this period, the average progesterone content increased by more than 5% in 56% of HF cows and 68% of SIM cows (group A). The average increase in P4 content was 1.37 ng/mL (average increase of 8.94%) in HF cows and 2.32 ng/mL (average increase of by 15.01%) in the SIM group. The cows in which the increase in progesterone content (P4_29-31_) did not exceed 5% (group B) included 44% of HF cows (average decrease of −0.53 ng/mL) and 32% of SIM cows (average decrease of −0.69 ng/mL). Compared to the initial value (P4_29_), the average progesterone level decreased by 3.89% in HF and by 4.85% in SIM.

Cows in which the average progesterone content increased by more than 5% between days 29 and 31 (group A) had a lower rate of lipolysis (Table 3) Cows with weaker production, in which P4_29-31_ did not exceed 5% (group B), were characterized by more intensive lipolysis (NEB markers). The milk of the latter group had a higher proportion of C16:0 (by 1.3%; *p* ≤ 0.05) and C18:1 (by 0.44%.; *p* ≤ 0.05). In the case of C18:0 this tendency was noted only in SIM, in which the difference was 0.34% (*p* ≤ 0.05). An increased share of C18:2, confirmed by a difference of 0.12% (*p* ≤ 0.05), was noted only in HF (Table 3). This tendency was confirmed by the negative values of the correlation coefficient, ranging from −0.326 to −0.712 (*p* ≤ 0.05).

More intensive release of NEFA during NEB was associated with lower P4_31-29_ in the milk (group B). Compared to cows with higher progesterone production (group A), the average NEFA content decreased by 24.0 mmol L^−1^ (*p* ≤ 0.05) in the case of HF and by 11.6 mmol L^−1^ in SIM (*p* ≤ 0.05). The correlation coefficient was −0.540 (*p* ≤ 0.05). The average BHBA and leptin levels of cows with higher average progesterone levels (P4_29-31_; group A) were lower by 0.111 mmol L^−1^ and 0.158 ng mL^−1^, respectively (*p* ≤ 0.05). However, a significant relationship was shown for the GLU level, confirmed only in the case of SIM (Table 3). Earlier ovarian cyclicity, indicated by higher average P4_29-31_ levels, was found in cows with higher GLU levels (group A). Compared to the cows with the lowest P4 levels, the differences were 0.21 mmol L^−1^ (*p* ≤ 0.05) in SIM and 0.13 mmol L^−1^ in HF. This tendency is confirmed by the positive correlation coefficient of 0.661 (*p* ≤ 0.05). Cows with lower values for NEB markers and higher average progesterone levels (P4_29-31_) usually showed ovarian cyclicity earlier (Table 3). In production practice, these cows had shorter calving-to-first-service intervals (CFSI) and calving-to-conception intervals (CCI). In the HF breed the differences between groups A and B amounted to 18.6 days for CFSI and 24.2 days for CCI (*p* ≤ 0.05), while the corresponding differences in SIM cows were 16.7 and 22.1 days (*p* ≤ 0.05). These tendencies were confirmed by the negative correlation coefficients of −0.491 (CFSI) and −0.536 (CCI), *p* ≤ 0.05.

## 3. Discussion

After calving, lactogenesis dramatically increases the need for energy. An energy deficit (NEB) at this time initiates changes in energy acquisition from the body’s stores [14,21]. The main source of energy to correct the NEB is lipolysis of fat deposited in the adipocytes. The fairly strong interaction between the rate of lipolysis and NEFA release and their use by the liver and other tissues suggests that the physiological adaptation of cows to NEB is an important factor in mitigating its consequences [5,22,23]. These studies correspond with our results, which may suggest that the effectiveness of NEFA utilization in the body may be linked to the potential of the breed. In the first stage of developing lactogenesis, release of NEFAs was greater in SIM, while the level of BHBA was lower. This may be explained by the stronger body condition of SIM cows at the start of lactation, as demonstrated by Heuer et al. [24]. However, the lower level of BHBA shown in the SIM cows in our study may indicate more efficient utilization of metabolites of lipolysis in the period approaching peak lactation. 

Lucy et al. [25] explained that the intensity of lipolysis during NEB may be a factor excessively straining liver metabolism, mainly due the level of NEFA metabolites reaching the liver. The authors showed a higher rate of lipolysis in breeds with high production potential, such as HF. This might explain the higher levels of C16:0 and C18:1 released from fat reserves during lipolysis in the milk of HF cows in our study. However, it should be noted that our results were obtained in a smaller population of cows. Such a relationship was also observed by Hellmuth et al. [26] and Walker et al. [9], who found that a higher rate of lipolysis was accompanied by an increase in the proportion of long-chain fatty acids, including C16:0 and C18:0. This is confirmed by our findings indicating that the shares of these milk fatty acids were positively correlated with DMP and LBSC. 

Some changes on the lactogenesis-lipolysis axis have a physiological cause, associated with uptake of substrates for lipogenesis from the digesta, and can be linked to greater production potential. Look and Garnsworthy [27] indicate that differences between breeds such as HF and Simmental may arise in part due to the higher rate of de novo fatty acid synthesis involving acetyl-CoA. Harvatine et al. [28] showed that intestinal uptake of the β-lipoprotein fraction could have ramifications for synthesis of C18:1 in the udder. On the other hand, Look and Garnsworthy [27] showed that NEB can reduce direct absorption of the cis-9 C18:1 fraction in the intestine and limit processes involving desaturase. Rukkwamsuk et al. [29] showed that this mechanism may be weakened by intensive production and inadequate availability of glucose. In our study, this tendency was confirmed by the negative DMPxGLU correlation. In addition, we showed that lower GLU content was accompanied by increased percentages of C18:1 and C18:0, which were also positively correlated with DMP. We are aware that the R values obtained in our study may be influenced by the small size of the populations analysed. On the other hand, it would be difficult to minimize stress in high-yielding herds with a larger population of cows. 

An important aspect of evaluating cows during lactation is body condition (BCS). Walker et al. [9] showed a strong association between the amount of fatty acids deposited in the adipocytes (stronger BCS) and their release during lipolysis, confirmed by higher percentages of C18:1 and C18:0 in the NEFA structure. In our study, we noted lower levels of NEFA and of C18:1 and C18:0 in individuals with less intensive lipolysis (LBCS) and in individuals beginning lactation with a stronger BCS (SIM). Vanbergue [30] also reported higher levels of these acids in the milk of cows with higher production potential (Holsteins). The milk of Normande cows, whose daily yield ranged from 7 to 12 kg, had lower content of MUFA, including from 2.5% to 7.5% lower cis-9 C18:1. The smaller share of cis-9 C18:1 in the milk of Normande cows was also explained by lower mobilization. The tendency observed in our study was less pronounced, but the cows with lower production potential (SIM) and in the period of less intensive lactation had a smaller loss of BCS and a lower level of C18:1. Delaby et al. [31] showed that the same energy level in the diet caused a more severe loss of BCS (−0.95) in cows with high production potential (Holstein) than in the Normande breed (−0.55). The authors also reported higher NEFA content at 20 and 60 days of lactation in Holstein cows. At 60 days, however, they also noted a lower glucose level in Normande cows fed a low-energy diet, but these cows had a higher overall pregnancy rate of 88.7%, compared to 73.4% in Holsteins. 

Lemley et al. [8] point out that a negative energy balance resulting from production potential and metabolic changes can affect the activity of cytochrome and aldo-keto reductase secreted in the liver. This may reduce the concentration of progesterone (P4) by increasing its conversion to 21- and 20α-hydroxyprogesterone. This was confirmed by the correlation coefficients linking the activity of cytochromes (P450 2C and P450 3A) and aldo-keto reductase (AKR1C) to body weight (0.22–0.45) and energy balance (0.03 to −0.31). In the authors’ opinion, this was associated with the insulin level, which they confirmed by using maize starch as feed stimulating its secretion. They showed that energy intake in this form can increase the P4 concentration and lead to a higher percentage of maintained pregnancies in early development. The authors also found that a higher insulin concentration can affect the time of the first ovarian activity after calving. In our study, the composition of the diet of both breeds was similar, so the trends observed in the progesterone and GLU levels are explained mainly by production potential and the physiological capacity to cope with NEB. 

As in our study, results obtained by Vargová et al. [11] indicate that in addition to GLU, leptin is an important mediator of processes of energy homeostasis during the development of lactogenesis. According to Drackley et al. [10] and Dann et al. [32], GLU at this time is the main source of energy, not only for lactose synthesis but for progesterone secretion processes as well. Leptin, on the other hand, as a bioregulator of appetite, not only influences NEB but can indirectly affect fertility. As in the present study, Vargova et al. [11] observed that the leptin concentration showed a tendency to decrease during early lactation and was positively correlated with BCS. This is in agreement with the research results noted by Ehrhardt et al. [33]. In contrast, our study showed a negative correlation coefficient for leptin × LBCS. In our opinion, this is linked to the rate of lipolysis, which during intensive lactation radically decreases the amount of adipose tissue. This limits secretion of leptin, which reduces the susceptibility of tissues to the effect of insulin. This may have contributed to the fact that the glucose level in our study was lowest during the period with the most intensive lactation. A similar tendency and a negative correlation between leptin levels and BCS were reported by Holtenius et al. [12]. Lower leptin production suggests stronger appetite and possibly higher feed intake. In our opinion this is to some extent due to the adaptability of cows during NEB, since as lactogenesis develops, the decreasing leptin level is conducive to feed intake and limits the loss of BCS. This may contribute to a lower level of BHBA, which was shown in our study. 

Zarrin et al. [2] showed that the physiological adaptation of cows to the consequences of NEB may to some extent be due to partial correction of the glucose (GLU) deficit by energy derived from BHBA. The authors demonstrated this in the case of the immune system. Drackley et al. [10] showed an increased ability to convert propionic acids to GLU in the liver during NEB. In their opinion the conversion mechanism was stronger when the cows consumed more carbohydrates. This may explain the changes in the content of BHBA and glucose in our study, where the less intensive lipolysis in cows with lower production potential put less strain on the liver, as indicated by the lower levels of NEFA and BHBA. This was one of the factors conducive to an increase in the blood GLU level. In this regard, the physiological ability to mitigate the consequences of NEB is an important aspect of adaptation in cows. These results correspond with research by Vargova et al. [11], which showed that excessive amounts of triglycerides released from the adipocytes impair hepatocyte performance. In addition, Drackley et al. [10] showed that this slows the gluconeogenesis pathway and may impair other mechanisms correcting the glucose (GLU) deficit. Such a deficit usually has an adverse influence on ovarian functions, associated in part with impairment of their secretion of progesterone. This was demonstrated by Vasconcelos et al. [34], who found that in postpartum cows a lower plasma GLU concentration was usually accompanied by a lower progesterone concentration. Studies by Radcliff et al. [35] and Taylor et al. [13] showed that GLU is an important mediator for liver receptors initiating the secretion of growth hormone (GHR-1A). This compound is important because its presence initiates production of insulin-like growth factor IGF-1, which co-regulates reproductive activity after parturition. According to Butler [5] and Gong et al. [36], weakened ovarian activity due to a low GLU level delays postpartum oestrus. This effect may be additionally increased by lower insulin and IGF-I concentrations, which according to Veronesi et al. [37] results from delayed formation of the luteal body, whose maturity is evidenced in part by the progesterone level. Research by Block et al. [38] suggests that the availability of energy (GLU) for reproductive functions may be partially linked to leptin levels. They showed that the leptin level was negatively correlated with GH and NEFA levels (−0.5) and positively correlated with glucose content (0.7). Our study showed a similar relationship between leptin and progesterone. These results correspond with the research by Block et al. [38] and indicate that developing NEB after parturition is responsible for the lower plasma level of leptin, which indirectly impairs the initiation of the luteal phase. We believe that a low leptin level is also responsible for suppressing functions that are not prioritized during early lactation (reproduction), mainly due to the mechanism of saving energy. According to Santos et al. [6], NEB can be considered the main factor impairing reproduction. This was confirmed by Waldmann et al. [39], who showed that maintenance of energy homeostasis contributes to earlier activation of the ovaries and a lower percentage of cows requiring repeated insemination. This indicates that earlier resumption of ovarian function reduces the risk of delayed conception. This was shown in the present study as well, as NEB markers were negatively correlated with progesterone levels. A higher progesterone level was associated with a shorter calving-to-first-service interval and calving-to-conception interval. The results of our study correspond with those reported by Kessel et al. [39]. The tendencies observed indicate that cows more often exhibit lower reproductive capacity when they undergo extreme metabolic changes during NEB, often manifested as high BHBA content and a severe loss of BCS.

## 4. Materials and Methods

### 4.1. Description of Herds

The research results were obtained during an experiment which was part of a larger project, called the Casein Programme, carried out in 2015–2020. The study was conducted in two dairy cattle herds. One breed was kept in each herd. The first herd comprised 70 Holstein-Friesian (HF) cows (N:51°56′55.34″; E:22°32′7.47″), and the other consisted of 40 Simmental (SIM) cows (N:49°23′21.88″; E:22°42′19.20″). The cows in the two herds constituted separate production groups. Cows whose milk production in the previous lactation was above average were included in the study. The criterion was established for each herd by calculating the modal value (Mo). The lactation yield of HF cows was 9890 ± 1803 kg of milk (mean ± SD), ranging from 6436 to 11,510 kg. The yield of SIM cows ranged from 6682 to 10,981 kg and was on average 8692 ± 1472 kg (mean ± SD). The Mo calculated for the herds was 10,059 kg for HF and 9426 kg for SIM. The criterion was met by 25 HF cows and 15 SIM cows. The cows included in the study were multiparous, with average parity of 3.1 ± 1.2 (HF) and 3.8 ± 1.6 (SIM). 

All cows were kept in free-stall barns in which the number of resting boxes and the length of the feeding area were adjusted to the number of cows. The barns were in compliance with animal welfare standards [40], and fans were used to ensure a comfortable temperature. Cows were milked in a milking parlour twice a day (at about 6.30 a.m. and 6.30 p.m.). The herds were kept under veterinary supervision.

### 4.2. Cow Diet

The cows included in the study were kept in separate production groups, where they were placed after calving. Their diet was established by analysing the levels of nutrients in the TMR (total mixed ration) components [41], approximate body weight (600 kg), and average milk production (35 kg/d, protein 3.3%, fat 4.1%). Nutrient requirements and balance were calculated based on standards for ruminants [42] and INRAtion 4.06 software (INRA, France). The nutrient balance and nutritional value of TMR in the herds is presented in Table 4. 

In both herds TMR was composed of the same ingredients (kg/cow): maize silage (22.5–25.2), barley (0.5–0.7), oats (0.5–0.7), wheat (0.5–0.7), triticale (0.9–1.4), rapeseed meal (0.7–1.1), soybean meal (2.0–2.4), NaCl (0.02), and chalk (0.1–0.2). Salt licks provided an additional source of microelements (NaCl—94.0%, Mg—0.20%, Co—0.18%, Zn—0.80%, Mn—0.83%, I—0.1%, Se—0.1%, water-insoluble compounds—4.0%). Diets were adjusted on an ongoing basis, mainly for protein content and energy balance, according to milk production, the milk protein and fat content, and the amount of uneaten feed, which was calculated as the average weight of uneaten feed for the group. Uneaten feed was weighed several times in each period, and the information was the basis for calculating average dry matter intake (DMI). 

Feed (TMR) was prepared in a feed wagon and supplied to cows three times a day (at intervals of about 8 h), using feed pushing. About 2 weeks before calving, the cows were given a preparatory diet. Cows had direct access to feed and water (open drinkers). 

### 4.3. Sample Collection and Analyses 

Milk and blood for analysis were collected in each of three stages of early lactation (SL): in the HF herd at 14 (I), 43 (II) and 68 (III) days of lactation and in the SIM herd at 17 (I), 45 (II) and 71 (III) days of lactation. Milk was sampled from morning and evening milking, individually from each cow, in the amount of about 250 mL. A pooled sample was used for analysis. Samples were stored at +4 °C until analysis. The representativeness of the samples was ensured by using a milk meter (DeLaval), which was also used to determine daily milk production. Blood was collected into tubes (Medlab-Products, Poland) intended for the type of analyses performed in this study and stored in refrigerated conditions. 

### 4.4. Indicators of Metabolic Changes and Negative Energy Balance (NEB) 

The occurrence of NEB was assessed on the basis of changes in body condition score (BCS), proportions of selected fatty acids (FA) in the milk, and levels of NEFA, BHBA, glucose (GLU) and leptin in the blood.

BCS was calculated as the average of two scores (by a feeding technician and a veterinarian). Cows were assessed on a 5-point scale [43]. The first BCS assessment was performed about 2 weeks before the expected calving date. It was on average 2.79 in the HF herd and 2.83 in SIM. Subsequent assessments were made on the days when samples were collected for analysis. The results were used to calculate the loss of body condition (LBCS).

The percentages (by weight) of fatty acids C16:0 (palmitoleic), C18:0 (stearic), C18:1-t9 (elaidic), and C18:2 (linoleic) were determined by gas chromatography with a mass detector (GCMS: Agilent Technologies Inc., Wilmington, DE, USA). Fat was extracted by the Röse-Gottlieb method [44]. Transmethylation of FA to methyl esters (FAME) was carried out in a block heater (ThermoFisher Scientific, Waltham, MA, USA) at 70 °C ± 0.5 [39]. Separation was carried out in a 100 m, 0.250 mm column (HP-88; SN:UST458414H, Agilent Technologies Inc., USA). Temperature program: injector 250 °C; furnace 95 °C (5 min), 120 °C (15 °C/min for 15 min), 210 °C (25 °C/min for 30 min), 250 (20 °C/min for 5 min). Carrier gas flow (H): 0.7 mL/min. Identification and percentages were based on retention times and standards (Supelco 37, No:47885-U; Sigma Aldrich, St Louis, MO, USA) Chemstation software (A09.03 Agilent Technologies Inc., Wilmington, DE, USA).Blood was drawn from the jugular vein before morning feeding. Levels of β-hydroxybutyrate (BHBA), non-esterified fatty acids (NEFAs) and leptin were determined in plasma. Blood was collected into tubes with a clot activator and refrigerated (+4 °C). Blood for GLU determination was immediately placed on ice (sodium fluoride). Plasma was separated by centrifuging (1500× *g* at 4 °C for 20 min) and stored at −75 °C until analysis. BHBA levels were determined using commercial Randox kits (Randox Laboratories Ltd., Crumlin, UK) and read on a UV-Vis spectrophotometer (Varian Inc., Palo Alto, CA, USA). Leptin content was determined by ELISA, using antibodies specific for cattle (EIAab, Wuhan, China). GLU was determined using a commercial Randox test (Randox Laboratories Ltd., Crumlin, UK) and a UV-Vis spectrophotometer (Varian Inc., Palo Alto, CA, USA). The NEFA percentage in the blood was determined based on the sum of acids C14:0 (myristic) C16:0, C18:0, C18:1-t9, and C18:2. The lipid fraction was extracted from the blood with a mixture of hexane and isopropanol [45]. Separation was performed by GCMS as described above.

### 4.5. Fertility Indicators

The rate of recovery of the ovarian cycle after calving was assessed as the average serum level of progesterone (P4). The hormone content was determined at 24 (P4_24_), 27 (P4_27_), 29 (P4_29_) and 31 (P4_31_) days postpartum, in cows that had not undergone artificial insemination. The purpose of the test was to detect how quickly ovarian cyclicity was resumed (the luteal phase) during NEB. A commercial immunoenzymatic assay for quantification of progesterone in cattle was used for the diagnosis (ELISA; Eurofins Abraxis, PA, USA). Cows in which the increase in P4 between 29 and 31 days (P4_29-31_) was at least 5% were classified as group A, and the remaining cows as group B. 

Fertility of cows was diagnosed on the basis of two parameters: the length (in days) of the calving-to-first-service interval (CFSI) and the calving-to-conception interval (CCI). 

### 4.6. Statistical Analysis of the Results

The results were analysed in Statistica 12.0. software (StatSoft Inc., Tulsa, OK, USA). Agreement with normal distribution was checked for all parameters (Kolmogorov–Smirnov test). Statistical analysis was performed within each breed group (HF and SIM). The grouping variables were the stage of early lactation (SL) and the category of changes in share of progesterone (groups A and B). Data were analysed using a multivariate model of canonical analysis and repeated measures GLM. The estimation of the correlation value was preceded by analysis of the consistency of the residuals with the normal distribution. For this purpose, an analysis of variance of the residual values was performed and R^2^ was calculated. The tables show correlations where R^2^ was greater than 0.5. Means (LSM), standard error of the mean (SEM), mode (Mo), and maximum and minimum values were used in the statistical analysis. A 95% confidence interval was adopted (*p* ≤ 0.05).

## 5. Conclusions

The results suggest that postpartum NEB can play an important role in delaying processes of the oestrous cycle. Intensive lactation resulting from genetic potential can be regarded as a factor that may have a significant adverse impact on reproductive performance, mainly due to induction of NEB and the resumption of reproductive functions during increasing lactation. The rate of triglyceride release depends on daily milk production, and therefore the adaptability of the liver should be considered an important element of mitigation of the consequences of NEB. The results obtained for mean progesterone content and early ovarian cyclicity resumption indicate that cows of the same breed may adapt in varying degrees to the consequences of NEB. These adaptive features can be used during selection of cows to extend their productive life, which is most often shortened due to deteriorating reproductive performance and metabolic problems. Although our research results were obtained in relatively small populations of cows, the tendencies observed are generally consistent with the findings of other researchers. This is worth noting because smaller-scale experiments conducted in production conditions can minimize stress in the herd. 

## Figures and Tables

**Table 1:** Changes in daily milk production (DMP), loss of body condition (LBCS), NEB biomarkers and correlations with DMP and LBCS in early lactation (3 stages).

Parameter	Holstein-Friesian (25)			Simmental (15)			SEM	Correlation (*p* ≤ 0.05) x	
	Stage of Early Lactation (SL)							DMP	LBCS
	I	II	III	I	II	III			
DL (day)	14 ^c^	43 ^b^	68 ^a^	17 ^c^	45 ^b^	71 ^a^	6.45	0.464	−0.795
DMP (kg/day)	23.5 ^c^	32.4 ^b^	36.9 ^a^	19.8 ^c^	28.8 ^b^	34.1 ^a^	3.24	0.772	-
LBCS (points)	−0.21 ^b^	−0.29 ^a^	−0.31 ^a^	−0.22 ^b^	−0.23 ^b^	−0.26 ^a^	0.08	-	0.772
C16:0 (%)	23.08 ^c^	26.18 ^b^	28.27 ^a^	21.58 ^c^	24.69 ^b^	26.63 ^a^	0.15	0.676	0.663
C18:0 (%)	11.98	12.02	11.94	11.43 ^c^	11.72 ^b^	12.04 ^a^	0.04	0.345	0.324
C18:1 (%)	1.81 ^b^	3.12 ^a^	3.58 ^a^	1.93 ^b^	2.83 ^a^	3.25 ^a^	0.03	0.431	0.552
C18:2 (%)	1.67	1.73	1.65	1.52 ^b^	1.85 ^a^	1.91 ^a^	0.02	0.354	0.338
NEFA (mmol L^−1^)	210.5 ^c^	262.7 ^b^	298.6 ^a^	176.4 ^c^	234.4 ^b^	272.8 ^a^	2.88	0.805	0.634
BHBA (mmol L^−1^)	0.638 ^c^	0.998 ^b^	1.424 ^a^	0.639 ^c^	0.918 ^b^	1.307 ^a^	0.02	0.810	0.789
Glucose (mmol L)	2.84 ^a^	2.33 ^b^	2.21 ^b^	2.92 ^a^	2.47 ^b^	2.38 ^b^	0.01	−0.415	−0.368
Leptin (ng ml^−1^)	2.92 ^a^	2.68 ^b^	2.59 ^b^	2.76 ^a^	2.83 ^a^	2.51 ^b^	0.02	−0.530	−0.507

^a,b,c^—*p* ≤ 0.05 (significance of differences within breed groups); NEB—negative energy balance; DL—day of lactation; DMP—daily milk production; LBCS—loss of BCS relative to BCS at 2 weeks before calving; NEFA—non-esterified fatty acids; BHBA—β-hydroxybutyrate.

**Table 2:** Changes in ovarian activity assessed on the basis of progesterone (P4) content in milk (ng/mL) on successive days.

Test Day	Holstein-Friesian (25)			Simmental (15)			SEM
	Mean	Min	Max	Mean	Min	Max	
P4_24_	4.71 ^c^	4.04	5.33	4.86 ^d^	4.07	5.42	1.15
P4_27_	6.61 ^b^	3.78	8.89	6.98 ^c^	4.73	8.45	1.19
P4_29_	14.44 ^a^	10.79	19.61	15.11 ^b^	10.61	20.47	0.89
P4_31_	15.34 ^a^	9.89	21.54	16.31 ^a^	9.48	22.70	1.29

^a,b,c,d^—*p* ≤ 0.05 (significance of differences within breed groups); DMP—daily milk production.

**Table 3:** Changes in indicators of NEB and fertility depending on classification to groups A and B according to the degree of changes in average progesterone (P4) levels between 29 and 31 days postpartum (P4_29-31_).

Parameter	Breed Groups					
	Holstein-Friesian		Simmental		SEM	Correlation (*p* ≤ 0.05) P4 x
	P4_29-31_ Classification					
	A	B	A	B		
Percentage of Cows (%)	56 (14/25)	44 (11/25)	68 (10/15)	32 (5/15)		
P4_29_ (ng/mL)	15.32	13.22	15.46	14.44	2.05	-
Change P4_29-31_ (ng/mL)	1.37 ^a^	−0.53 ^b^	2.32 ^a^	−0.69 ^b^	0.22	-
Change P4_29-31_ (%)	8.94 ^a^	−3.98 ^b^	15.01 ^a^	−4.85 ^b^	1.62	-
DPM (kg/day)	21.9 ^b^	25.6 ^a^	18.6 ^b^	22.2 ^a^	0.54	−0.658
C16:0 (%)	22.61 ^b^	23.63 ^a^	21.08 ^b^	22.66 ^a^	0.21	−0.712
C18:0 (%)	11.97	12.09	11.33 ^b^	11.67 ^a^	0.08	−0.405
C18:1 (%)	1.57 ^b^	2.09 ^a^	1.82 ^b^	2.17 ^a^	0.07	−0.451
C18:2 (%)	1.62 ^b^	1.74 ^a^	1.53	1.49	0.33	−0.326
NEFA (mmol L^−1^)	199.9 ^b^	223.9 ^a^	175.3 ^b^	186.9 ^a^	3.9	−0.540
BHBA (mmol L^−1^)	0.569 ^b^	0.727 ^a^	0.619	0.689	0.01	−0.430
Glucose (mmol L^−1^)	2.76	2.63	2.98 ^a^	2.77 ^b^	0.03	0.661
Leptin (ng ml^−1^)	0.328 ^a^	0.217 ^b^	0.307	0.279	0.04	−0.300
CFSI (days)	43.8 ^b^	62.4 ^a^	42.9 ^b^	59.6 ^a^	1.3	−0.491
CCI (days)	66.6 ^b^	90.8 ^a^	65.7 ^b^	87.8 ^a^	2.1	−0.536

^a,b^—*p* ≤ 0.05 (significance of differences within breed groups); LBCS—loss of BCS relative to BCS at 2 weeks before calving; DMP—daily milk production; NEFA—non-esterified fatty acids; BHBA—β-hydroxybutyrate; CFSI—Calving-to-first-service interval; CCI—Calving-to-conception interval.

**Table 4:** Nutritional value and nutrient balance of the diet during early lactation in Holstein-Friesian (HF) and Simmental (SIM) cows.

Parameter	HF (25)	SIM (15)
Number of cows	(25)	(15)
Dry matter (%)	42.4	41.5
Protein (%)	16.4	15.4
Fibre (%)	19.2	19.1
Fat (%)	2.5	2.2
Ash (%)	8.0	7.7
Starch (%)	22.7	22.8
ADF (%)	22.8	22.4
NDF (%)	39.5	38.7
peNDF (%)	30.6	30.9
UFL	21.5	20.05
PDIN (g)	2459	2318
PDIE (g)	2201	2097
Energy (MJ NE_L_ *):		
Requirement	151.8	128.2
Intake	153.3	130.1
Balance	+1.5	+1.9
DMI (kg/day)	23.1	24.4

ADF—Acid detergent fibre; NDF—Neutral detergent fibre; peNDF—Physically effective; UFL—feed unit for milk (1700 kcal NE_L_); PDIN—protein digested in the small intestine, calculated from feed nitrogen (N) available in the rumen; PDIE—protein digested in the small intestine, calculated from feed energy (E) available in the rumen; *—1 MJ = 1184 Mcal, DMI—Dry matter intake.

## Data Availability

Available from the corresponding author. Because of the participant consent obtained as part of the recruitment process, it is not possible to make these data publicly available.
